# 

**Published:** 1930

**Authors:** 


### CONTENTS.

| | Page |
|---|---|
| The Long Fox Memorial Lecture: The Influence of Food on the Production and Prevention of Disease. By J. A. Nixon, C.M.G., M.D., F.R.C.P. | 255 |
| The Beddoe Memorial Lecture: Anthropology: Old and New. By Sir Arthur Keith, F.R.S. | 287 |
| Notes on the Treatment of Eclampsia. By G. H. Temple, M.B., C.M. | 307 |
| Five Cases of Injury to Main Vessels occurring in Civil Practice. By Robert V. Cooke, M.B., Ch.M., F.R.C.S. | 311 |
| Perforation in Paratyphoid B. By Frank Bodman, M.B., M.R.C.S., and Paul Bodman, M.B. | 317 |
| A Case of Typhoid Fever with Co-existent Bacillus Coli Infection. By J. Innes Noble, M.B., Ch.B., F.R.C.S. | 323 |
| Reviews of Books | 331 |
| Editorial Notes | 339 |
| Obituary: James Paul Bush, C.M.G., C.B.E., Ch.M. | 342 |
| Meetings of Societies | 346 |
| The Medical Library of the University of Bristol | 350 |
| Local Medical Notes | 351 |

